# Clinical study protocol on electronic cigarettes and nicotine pouches for smoking cessation in Pakistan: a randomized controlled trial

**DOI:** 10.1186/s13063-023-07876-y

**Published:** 2024-01-02

**Authors:** Abdul Hameed, Daud Malik

**Affiliations:** Department of Research, Alternative Research Initiative, Islamabad, Pakistan

**Keywords:** E-cigarettes, Nicotine replacement therapy, Smoking cessation, Tobacco control, Tobacco harm reduction

## Abstract

**Background:**

Pakistan is one of most vulnerable low- and middle-income countries with 29 million adult active tobacco users. Smoking cessation services are lacking as the tobacco control initiatives have largely failed to address the smoking endemic. Over the last 5 years, Pakistan has witnessed the use of innovative tobacco harm reduction (THR) products such as e-cigarettes and nicotine pouches. However, their use remains limited. THR products are imported legally as consumer goods and are taxable. The lack of sufficient data for THR and its application is a challenge in gauging their effectiveness in assisting smokers quit combustible smoking. Evidence-based studies can help in measuring the effectiveness of e-cigarettes and nicotine pouches as smoking cessation aids.

**Method:**

Keeping in view the study objectives, a sample size of 600 participants will be sufficient to assess the effectiveness of e-cigarettes and nicotine pouches for smoking cessation in Pakistan. Of these, 200 participants each will receive e-cigarettes and nicotine pouches along with basic care counselling, while the remaining 200 participants will only receive basic care counselling for 48 weeks. The association of participants’ characteristics with smoking and health status will be based on the bivariate and multivariate analysis. The simple *t*-test and variance analysis will assess the differences in intervention indicators between the control and treatment groups. For the inferential analysis, the average treatment impact will be based on the quasi-experimental techniques such as difference in difference (DID) or propensity score matching (PMS).

**Discussion:**

The study will evaluate the participants at the baseline as they decide the quit date. After every 12 weeks, a follow-up survey with the participants will be conducted. Results are anticipated to inform the public, decision-makers, and researchers about the effects of using e-cigarettes and nicotine pouches in the short- and medium-term periods. Critically, the potential of e-cigarettes and other alternative nicotine delivery systems as smoking cessation aid will be assessed.

**Trial registration:**

ClinicalTrials.gov NCT05715164. Registered on February 6, 2023. Protocol version. Protocol version 1.0, 14-12-2022

Trial in progress and not yet recruiting participants. Estimated primary data collection date—April 2024

**Supplementary Information:**

The online version contains supplementary material available at 10.1186/s13063-023-07876-y.

## Introduction

There are more than a billion consumers of higher risk tobacco products worldwide, including cigarettes, bidis, and cigars. The World Health Organization (WHO) estimates the tobacco pandemic kills more than eight million people annually [1]. In LMICs, where the burden of smoking-related illness and mortality is highest, the majority of the world’s 1.1 billion smokers reside [2]. With 29 million active adult tobacco users, Pakistan is one of the most vulnerable LMICs in South Asia. More than 45% households in Pakistan use tobacco [3].

Estimates say tobacco kills around 163,600 people each year in Pakistan. Secondhand smoke is responsible for over 31,000 of these deaths [4]. Annually, on average, 82 billion cigarettes are consumed [5]. A total of 16.8 million adults who work indoors and 56.3 million at home are exposed to secondhand smoking [6]. The total cost of all smoking-related illnesses and fatalities in 2019 was Rs615.07 billion (US $3.85 billion). However, the Rs120 billion collected in taxes from the tobacco industry in 2019 covered only 20% of the overall costs associated with smoking [7].

Pakistan’s tobacco control initiatives have focused on restricting or disrupting the demand for cigarettes, with little emphasis on cessation. The country has employed various initiatives, such as cessation clinics, public awareness campaigns, and restrictions. However, the number of smokers has grown over time. The efficacy of such therapies is questionable and requires factual research. Furthermore, information about cigarette cessation services is not readily available; few people are aware of this. Even well-educated young smokers who want to quit are unaware of the cessation programs [8]. Due to a lack of knowledge about cessation services, almost half of all tobacco cessation attempts in Pakistan are unassisted [9].

THR has garnered a lot of attention as a potential smoking cessation aid globally. As a result, numerous international studies have been conducted to evaluate the efficacy of e-cigarettes with traditional cessation approaches and nicotine replacement therapy (NRT). To determine the effectiveness of e-cigarettes in helping people quit smoking, a number of randomized controlled trials (RCTs) were carried out using a variety of study designs and participant criteria. These studies included Lee et al. [10], Walker et al. [11], Smith et al. [12], Chiang et al. [13], Nancy et al. [14], Halpern et al. [15], Martinez et al. [16], Tseng et al. [17], and Janet et al. [18]. Results from these trials suggested comparable effectiveness between e-cigarettes and certain NRTs, counselling, and non-nicotine, indicating that e-cigarettes might offer a viable alternative for individuals attempting to quit smoking. However, findings also highlighted the importance of additional research to understand the broader impact of e-cigarettes on both individuals and populations, especially considering potential side effects and long-term consequences.

Recent studies on barriers to smoking cessation suggest that the social interaction and friendships at work, home, and in public are the primary motivators for initiating smoking. The main deterrent to obtaining medical help to quit smoking seems to be lack of knowledge about its availability. The knowledge regarding THR, particularly e-cigarettes, can be described as imprecise. In Pakistan, tobacco control initiatives lack focus on quitting smoking. To help people stop smoking, medical and professional support is a must. This support should be provided with education campaigns about the harms of combustible smoking and the role of THR in smoking cessation [19, 20]. On the other hand, both the public and health professionals have little knowledge regarding nicotine. More than two-thirds of doctors (70%) in Pakistan think nicotine causes cancer. With the use of research-based therapies and communication strategies, misconceptions about nicotine can be corrected [21].

THR is barely making its presence felt in Pakistan. Individuals and business owners of the THR products remain wary of any possible regulations/rules that may delay or shut down their businesses. There are no clear or defined rules and regulations governing the use of THR, including import, manufacturing, or product content. E-cigarettes and other THR products are legally imported as consumer goods and subject to taxation. The availability of data regarding THR products and their use is limited. According to some estimates, the number of THR users in Pakistan is somewhere between 30,000 and 50,000 with unreliable evidence of dual use of conventional smoking and vaping. Most of the vaping outlets in Pakistan are in the upscale localities of major cities such as Karachi, Lahore, Rawalpindi, and Islamabad.

In this context, this study proposes a randomized controlled trial and primary data from two metropolitan districts — Islamabad and Rawalpindi — to examine the effectiveness and role of electronic cigarettes and nicotine pouches for smoking cessation in Pakistan. This would be the first nationwide clinical investigation into the effectiveness of nicotine pouches and e-cigarettes. This study is important because it looks specifically at the use and efficacy of nicotine pouches and e-cigarettes for quitting smoking in Pakistan. Numerous statistics demonstrate that Pakistan bears a heavy burden of tobacco-related sickness and death. The tobacco control policies have mostly focused on reducing the market for cigarettes, with little attention on programs for quitting. The number of smokers has increased in spite of numerous efforts. This important knowledge gap will be filled by the study through the implementation of a randomized controlled experiment. The study intends to produce empirical evidence that could potentially inform laws, regulations, and healthcare strategies suited to the Pakistani population and globally by performing the first nationwide clinical investigation on the efficacy of these items. With its thorough approach, it intends to add to the body of knowledge already available on smoking cessation therapies while also attempting to shed light on the practical consequences of employing THR products for quitting in developing countries.

The section on literature review presents the theoretical and empirical review about e-cigarettes and nicotine-related interventions, sample, methodology, and results. The “Materials and methods” section describes the data and its sources as well as the methodology. The “Discussion section” describes the THR scenario in terms of policy and regulation. The last section encapsulates the conclusions.

## Literature review

### Theoretical review

The act of burning tobacco and inhaling smoke is referred to as smoking tobacco. It is believed that pipe use began in Mesoamerica and South America from 5000 to 3000 BC. The burning of tobacco produces nicotine and other hazardous chemicals including nitrogen, carbon monoxide, and tar. As an addictive chemical, nicotine’s addiction depends on the way it is consumed. Cigarettes remain the dominant global nicotine delivery vehicle, and the global nicotine ecosystem is highly concentrated. However, the main risks associated with cigarettes are the chemicals produced during combustion, instead of nicotine, which makes them dangerous. Other compounds in smoke, such as tar, tobacco-specific nitrosamines, and benzene, primarily cause smoking-related diseases.

#### Biological aspects

A cigarette produces around 6000 chemical particles, which are the main cause of risks to human health [22]. Many studies show smoking kills millions of people worldwide every year. Smoking is the cause of diseases of the heart, lungs, mouth, stomach, and brain [23]. It also affects male and female fertility, sperm count, and mobility and increases the probability of miscarriage [24]. A recent study shows smoking is the leading cause of coronary heart disease, which affects people of all ages [25]. Another indigenous study reveals tobacco and clinical tuberculosis together account for half of adult male fatalities from tuberculosis between the ages of 25 and 69. This shows they lose, on average, 20 years of life expectancy, which is crucial for the economy [26]. The results of another 50-year-old follow-up study show two out of three smokers who start smoking early die from smoking-related causes. The likelihood of dying because of smoking was extremely high [27]. Numerous gases and particles as well as minute amounts of radioactive particles are released when people smoke tobacco. These gases and particles enter lungs, travel through blood circulation, and affect every body organ, impairing their functionality, including the lungs, brain, heart, kidneys, and stomach. This causes high blood pressure, irritate the lining of bronchial tubes, harm the inner walls of arteries, platelets, and abdomen aortic aneurysm, along with clots and blockages, decreased oxygen and blood flow, and the slow movement of lung’s cilia [28, 29].

#### Psychological aspects

The most crucial factors in determining what prevents a person from quitting smoking are smoking behavior and personality traits [20, 30]. Most tobacco or cigarette users think smoking helps them cope with anxiety, stress, and depression [31]. According to some studies, smokers puff cigarettes to reduce negative emotions and prevent unfavorable emotional disorders [32]. The relationship between smoking and psychological issues such as depression, stress, and anxiety is strong [33]. Most research indicates in this regard smokers have their own beliefs and presumptions. According to a study [34], smokers claim they smoke to relieve stress and enjoy themselves. Others argue smoking eases mental discomfort and calms the mind [35]. Though most smokers are confident about quitting when they want to, they are unable to give up the habit [20]. However, studies show that moderate and heavy smokers experienced higher levels of anxiety and depression compared to nonsmokers [36]. Social connections are crucial for both physical and psychological health. Even under extreme stress, empathy aids in managing emotions and feelings and encourage helpful behaviors [37], and one of the factors influencing smoking behavior is a lack of empathy [38].

#### Social and cultural aspects

The most crucial factors in implementing the smoking cessation initiatives are social and cultural characteristics of the individual and the community. A variety of social and cultural context-related factors influence smoking behavior [39]. A significant obstacle to quitting smoking is social acceptance of its risks [20]. Numerous studies highlight the lack of properly implementing tobacco control policies as one of the causes for the societal acceptance of smoking in public areas, workplaces, educational and healthcare institutions, and in local communities [40]. A study highlights [41] young smokers as “social smokers,” who exhibit less desire to give up smoking. These groups are influential in leading other teenagers and youngsters to smoking. Peer pressure is commonly acknowledged as a critical element in initiating young people’s early nicotine experimentation and their decision to continue smoking [42].

In numerous cultures, the tobacco plant, and its byproducts, such as snuff and cigarettes, is also meaningful. Across cultures, tobacco use has different connotations. Smoking has a direct relationship with culture and religious practices. Most religions, including Christianity, Judaism, Buddhism, Islam, and Hinduism, have anti-smoking stances, and those who participate in religious activities tend to smoke less frequently [43]. In many countries, men are more likely than women to smoke. These social and cultural factors affect how a government chooses to implement tobacco control policies.

#### Economic aspects

Smoking has a significant negative economic impact everywhere. The economic cost is classified into three categories — direct, indirect, and intangible. The cost of smoking-related diseases is assessed using a direct approach, and the indirect cost is determined by the loss of productivity and the absenteeism of smokers owing to smoking-related illnesses. The intangible costs cannot be easily quantified, such as loss of life and the burden of pain and suffering caused by smoking-induced illness [44]. Several studies have also calculated the economic cost of smoking in developed nations. In developing nations, there is little concrete information or documentation regarding the economic cost of smoking. However, the burden of noncommunicable diseases (NCDs) is significant in developing nations. NCDs result in decreased productivity and income at the household level. At the national level, smoking-related illnesses result in economic and productivity losses and are estimated to be in billions of dollars annually [45]. The other indirect economic cost is the spending to reduce smoking prevalence. The developed and developing countries spend billions of dollars on the tobacco control programs [46].

#### Nicotine, tar, and carbon monoxide

Michael Russell, who is considered the father of tobacco harm reduction, said: “People smoke for the nicotine, but they die from the tar.” One of the first researchers to identify nicotine as the primary reason smokers become addicted, Russell, was an early developer of and advocate of nicotine replacement therapy (NRT). He came up with the idea medium and high nicotine, low tar cigarettes [47]. Tar paralyzes and can eventually kill cilia in the airways, and when damaged, the toxins in tar can travel deeper into lungs. Eventually, this can result in emphysema, bronchitis, and lung cancer. The toxins can be carried into the bloodstream and begin moving to other parts of body and damage the heart, brain, and stomach. Carbon monoxide is a poisonous gas that takes the place of oxygen in blood. This forces heart to work much harder and stops lungs from working properly. Cells and tissues are prevented from getting the oxygen they need. This can lead to heart disease and stroke [48].

### Empirical review

A chronological and systematic empirical review has been employed to understand the previous research structure. These included the goals of the study, the testing of hypotheses, the use of data, the primary and secondary outcomes, the participant eligibility standards, the methodological framework, statistical analysis, and variable associations and factual findings. Authors first searched and evaluated clinical studies on smoking therapies to control smoking or lower the relative risk of smoking by using tobacco harm reduction products such as e-cigarettes, nicotine patches, public awareness campaigns, and other NRTs.

The authors carefully evaluated the search results’ titles and/or abstracts, and any papers that were not relevant were eliminated. The authors independently acquired full copies of the remaining studies and evaluated them to ensure they matched all inclusion criteria such as study topic, participants, interventions, and outcome measures. Where needed, the authors were approached for more information to help with the decision-making process with regard to irrelevancy or lacking sufficient information. As a first step, 20 potential studies pertaining to various interventions for controlling and quitting smoking were selected. The selection of the studies, published between 2015 and 2020, was based on a sampling design, methodological framework, and analysis.

Out of the 20 studies on e-cigarettes and NRTs, eight were conducted in the USA; three in New Zealand; two in the UK; two each in Italy, Canada, and Australia; and one in South Korea. These studies have been conducted in the developed countries where the health institutions are well equipped and administered efficiently. On the other side, the populations of developing nations have limited access to modern, well-run healthcare facilities. Understanding tobacco harm reduction, especially the role of e-cigarettes and NRTs in quitting smoking, is still lacking in these countries. Additionally, healthcare research and development are also lacking. Mostly studies used two-arm study design. However, seven studies used three-arm study design. All the selected studies have been published in renowned journals.

#### E-cigarettes with NRT intervention

A randomized trial of e-cigarettes for quitting smoking conducted by a study [49] used a three-arm design — e-cigarettes with nicotine, nicotine patches, and placebo e-cigarettes. The criteria for eligibility included at least 18 years old, smoking 10 or more cigarettes per day (CPD) for the previous year, wanting to quit, and being able to give consent. The main result was quitting smoking for seven days. The follow-up periods were 1, 3, and 6 months. The treatment and the control groups were compared with multivariate regression adjustment and the *χ*^2^ test. According to the findings, e-cigarettes, whether they included nicotine or not, were only slightly more efficient than nicotine patches at aiding smokers in quitting. A few negative side effects were also noticed. E-cigarettes’ role in tobacco control was found to be unclear, with additional research recommended to clarify their overall advantages and disadvantages, both for individuals and for populations.

To determine the effectiveness of e-cigarettes and NRT for smoking cessation, another study [50] conducted a two-group pragmatics multicenter RCTs. The first group received nicotine replacement treatment, while the second group was given cigarettes. To examine the effects of e-cigarettes and NRT, a total of 884 subjects were analyzed using follow-up technique. The primary objective was sustained abstinence for 1 year. It was confirmed biochemically and assessed using a mixed-method analysis with binary regression and the generalized linear method (GLM), while secondary outcomes included participant-reported treatment use and respiratory symptoms. E-cigarettes were found to be more effective method of quitting smoking compared to NRT. Lee et al. [10] conducted a two-arm, single-center RCT to examine the efficacy of e-cigarettes and NRT for smoking reduction and cessation. Male adults over 18 who had smoked for at least 3 years and who were motivated to quit smoking altogether or cut back on their cigarette intake were qualified as eligible subjects if they had smoked at least 10 CPD in the year prior.

The 9- to 12-week and 9- to 24-week continuous abstinence rates served as the primary outcomes, and the 7-day point prevalence of abstinence at weeks 12 and 24 was the secondary outcome. The primary and secondary outcomes of the interventions were determined by a mixed analysis with independent *t*-test, Fisher-Freeman-Halton extension of Fisher’s probability test, and logistic regression analyses. The results of 150 participants revealed that nicotine gum and e-cigarettes had comparable effects on quitting smoking. E-cigarettes were also well tolerated by the study participants. Consequently, using e-cigarettes as an NRT to quit smoking may be a good option. Bonevski et al. [51] conducted a pilot RCT comparing nicotine vaping products to NRT for smoking cessation. Under the pragmatic two-arm design, one group received NRT control plus telephone Quitline behavioral assistance, and the other group received nicotine vaping product plus telephone Quitline behavioral support.

The study was open to adults aged 18 and up who were tobacco users at the time of enrollment and had the competence to provide informed consent. The primary outcome was 7-day point prevalence abstinence with no more than five cigarettes since the date of quit. At baseline, the secondary outcomes quitting self-efficacy, motivation to quit, and the heaviness of smoking index were examined. To compare the outcome responses between the treatment and control groups at the 6- and 12-week follow-up strategies, the study employed mixed-effect models with logit and generalized linear regression conical link functions. An analysis of 63 respondents showed NRT combined with Quitline counselling was more appealing for quitting smoking. Participants who reported decreased cigarette cravings, decreased perceptions of withdrawal symptoms, and decreased cigarette smoking reported using both nicotine vaping products and NRT.

Walker et al. [11] carried out a three-arm pragmatic RCT with nicotine patches, nicotine e-cigarettes, and nicotine-free e-cigarettes. A total of three groups were given nicotine patches, nicotine patches and nicotine e-cigarettes, and nicotine patches and nicotine-free e-cigarettes, respectively. The qualified requirements were a current smoker who was at least 18 years old and wanted to stop smoking. The primary outcome was exhaled carbon monoxide (CO)-verified continuous smoking abstinence 6 months after the agreed quit date measurement with a Bedfont Smokerlyzer with a reading of nine ppm or lower sign. The secondary outcomes included continuous abstinence at 12 months using 7-day point prevalence abstinence (no cigarettes, not a single puff, in the previous 7 days) technology at quit date, 1, 3, and 6 months after the agreed quit date. This study used quit rates, relative risks, and risk differences at 95% confidence intervals for both the treatment and the control groups. Overall, 1124 participants received interventions. Using nicotine patches along with a nicotine e-cigarette can modestly improve smoking cessation results compared to using patches along with a nicotine free e-cigarette or using patches alone, with no signs of short-term harm.

Smith et al. [12] two-arm RCT gave e-cigarettes to one group and NRT to the other group. Smokers with a history of unsuccessful quitting attempts and no preference to use NRT or e-cigarettes were included as the participants for the study. The primary goal was a minimum 50% reduction in cigarette intake that was biochemically validated at 6 months, and the secondary outcome was sustained abstinence validated at 6 months. The less than 8-ppm CO level was utilized. To calculate the relative risk of e-cigarettes against NRT, binomial regressions were carried out using the generalized linear model with binomial distribution and logarithmic link. When the parametric assumptions were not met, Wilcoxon’s signed-rank tests compared the differences in product ratings and CPD between research arms. The analysis of all 135 participants revealed that e-cigarettes were superior to NRT in facilitating validated long-term smoking reduction and cessation.

#### E-cigarettes with counselling

To determine the effects of e-cigarettes and smoking cessation in pregnant women, Chiang et al. [13] conducted a two-arm RCT. The first group received cigarettes and text messages, while the other group received e-cigarette with dual use of cigarettes and text messages. The eligibility age was 14 years or older, currently pregnant, possesses a smartphone, willingness to receive texts, and smoked at least 15 cigarettes in the past 2 weeks. The impact of e-cigarettes on CPD was the primary outcome. Smoking cessation at the 7-day period of abstinence was the secondary outcome. The effect of e-cigarettes was investigated with the simple mean difference values. The results of this pilot study with 471 participants showed that e-cigarette’s impact on smoking behaviors among pregnant women in the USA is mixed, and relative effect of e-cigarettes on smoking reduction should be examined in future.

A study by Nancy et al. [14] examined the relationship between e-cigarette use and smokers quitting smoking after a hospitalization. One of the groups received regular standard care, whereas the other received regular care plus an intervention. The main result was the use of e-cigarettes in the first 3 months following discharge, with tobacco abstinence measured at 6 months using biochemically validated measures. To calculate the intervention impact of abstinence, propensity score matching (PSM) approaches were employed. The analysis of 1100 participants revealed interesting facts. Over 25% of smokers who were trying to quit used e-cigarettes for 3 months. When compared to smokers who did not use e-cigarettes regularly, this pattern of e-cigarette usage was related with a lower 6-month rate of tobacco abstinence. Further research is necessary to determine whether routine use of e-cigarettes promotes or inhibits smoking cessation.

The study by Halpern et al. [15] looked at e-cigarettes, rewards, and medications for cessation with a pragmatic trial. Usual care, free e-cigarettes, an incentive plus free cessation aids, and a redeemable deposit plus free cessation aids were the five implemented interventions. The biochemical proof of urine sample with a cotinine level of less than 20 ng per milliliter was the main technique for verifying abstinence. The impact of the interventions was determined by using the logistic regression analysis. Financial incentives increased the rate of maintained smoking cessation compared to free cessation aids. To better understand the short-term effects of e-cigarettes with high smoking-related risk, Masiero et al. [52] opted for ca double-blind, three-arm RCT with standard care, e-cigarettes, and standard care, as well as a placebo e-cigarette. The eligibility requirements included at least 55 years old, smoking an average of 10 CPD or more for at least in the last 10 years, and not being engaged in any other quit program.

The primary objective was a change in pulmonary function (dry cough, shortness of breath, mouth irritation), while the secondary outcomes were a change in daily cigarette consumption and a change in the carbon monoxide concentration of expired air. Group comparisons were made based on nonparametric statistics. To analyze differences in respiratory symptoms, e-cigarette side effects, and any other categorical factors, a chi-square test was specially applied. To assess statistical differences in cigarettes, the Mann-Whitney *U*- (for two samples) and Kruskal-Wallis *H*- (for three samples) tests were utilized. The findings showed that e-cigarettes helped participants quite smoking and reduced CPD rate significantly decreased compared to the baseline values. This was valid for smokers who were prepared to stop smoking, but it can also be helpful for less motivated smokers taking part in clinical settings.

To test the efficacy of counselling, e-cigarettes plus counselling, and non-nicotine e-cigarettes plus counselling on smoking cessation, Eisenberg et al. (2020) [53] conducted three arms with a multicenter RCT. Adults who wanted to stop smoking and averaged at least 10 CPD were eligible. The point prevalence smoking abstinence was at 12 weeks after randomization, and the 7-day secondary end point was at 24 weeks, which were assessed at various follow-ups. Based on the binomial distribution, the pairwise comparison and risk differences with 95% confidence level were estimated. With logistic regression models, odds ratios were estimated. The analysis of data from 376 participants showed that for smokers motivated to quit smoking, the use of nicotine e-cigarettes in combination with counselling, opposed to counselling alone, significantly boosted point prevalence abstinence at 12 weeks. The differences were no longer significant at 24 weeks.

Martinez et al. [16] conducted a three-arm RCT to examine smoking cessation among dual users of combustible cigarettes and e-cigarettes. The first group received self-help booklets, while the second group received smoking cessation self-help booklets and monthly cessation materials, and the third group received dual user-specific booklets and monthly cessation materials. The eligibility requirements included at least 18 years old, smoking once a week or more in the prior year, and vaping once a week or more in the previous month. Across America, participants were enrolled using print, electronic, and social media forums. Self-reported 7-day point-prevalence smoking abstinence at each assessment point served as the primary outcome. Secondary outcomes were 7-day point-prevalence vaping abstinence and the cost per incremental smoking cessation. Generalized estimating equations and mixed analysis with univariate and multivariate logistic regression evaluated the effectiveness of interventions. Findings from a total of 2896 participants show that self-help information with e-cigarettes has a large potential for encouraging smoking cessation for those who use both combustible cigarettes and e-cigarettes.

#### E-cigarettes and NRT with nicotine and non-nicotine

Using a three-arm study design, Caponnetto et al. [17] conducted a 12-month randomized controlled trial to evaluate the effectiveness of e-cigarettes as a combustible smoking substitute. The study included e-cigarettes without nicotine, with 7.2 mg of nicotine, 5.4 mg of nicotine for the first two quarters, and 7.5 mg for the second two quarters. The eligibility requirements included being between the ages of 18 and 70, having smoked 10 cigarettes per day for the preceding 5 years, being in excellent health, and not actively trying to quit smoking or planning to in the next 30 days. The abstinence was examined with the carbon monoxide test. The main result was a 7-ppm CO concentration without smoking. For group comparison, nonparametric econometric tests were applied. There were 3 follow-up intervals: 12, 24, and 52 weeks. The findings support the claim that using e-cigarettes, both with and without nicotine, dramatically reduces the consumption of conventional cigarettes and results in long-lasting tobacco abstinence without having any negative side effects.

Tseng et al. [17] carried out a randomized control trial comparing the nicotine-containing electronic cigarettes with a placebo in terms of smoking cessation in young adults. The double-blind two-arm RCT had 99 subjects who were current smokers with greater than 10 CPD and validated greater than and equal to 8 ppm. The eligible age range was 21 to 35. The main result was a self-reported decrease in CPDs of more than 50% participants after three weekends of treatment. Multiple logistic regression analysis investigated the contribution of the predictors to the result of smoking reduction and repeated measure. ANOVA statistics examined variation within and between CPD rates. With the use of e-cigarettes, a diverse young adult sample of current, daily smokers who were not ready to quit were able to cut back on smoking. To determine the function of nicotine- and placebo-containing e-cigarettes in promoting reduction and subsequent cessation, more research is required.

With a double-blind three-arm study, Backer et al. [54] investigated the role of NRT in conjunction with low nicotine cigarettes for smoking cessation. The participants were smokers who had smoked an average of 15 cigarettes per day for at least 1 year before randomization and were between the ages of 21 and 65 years. Entry into the study required a carbon monoxide (CO) measurement of 15 ppm. The primary endpoint was 4 weeks of abstinence, which was established by self-report and verified by inhaled CO < 10 ppm for each individual, and continuous abstinence recorded from weeks 7 to 10. The intent-to-treat (ITT) and Fisher exact test evaluated hypotheses. To identify prognostic markers and conduct an adjusted analysis of the primary outcome, logistic regression was used. At the analysis level, 346 samples were obtained, including 114 for control and 232 for treatment groups. According to the trial findings, Quest brand of cigarettes plus NRT was more effective than active control plus NRT in attaining 4 weeks of continuous abstinence. There were no major side events associated with the investigational product. Walker et al. [55] assessed the combined effect of low nicotine cigarettes, NRT, and behavioral quit line care in a single-blind two-arm research. The participants were 18-year-old smokers interested in quitting smoking. The participants were not eligible if they were pregnant/breastfeeding, presently using NRT or non-cigarette tobacco products, had a stroke or angina in the previous 2 weeks, or were taking bupropion, clonidine, or other similar medications.

The primary outcome was smoking abstinence for 7 days, and the quit data was after 6 months. To analyze the treatment effect over time, repeated-measures analyses were also performed with generalized estimating equation (GEE) models. According to the findings, some smokers may be helped to quit by adding low nicotine cigarettes to the normal Quitline smoking cessation support.

By using a randomized control trial, Janet et al. [18] studied how to improve quit-and-win competitions to increase cessation among college smokers. The study had two groups: one with 615 participants in a multiple contest with no counselling and 602 individuals in a single contest with counselling. The minimum age to participate was 18. At the completion of the treatment, the 30-day point prevalence-verified abstinence after 6 months was the primary outcome. The secondary outcome was the same at 4 months. To determine how treatments affect the prevalence of smoking, the logistic and generalized linear mixed models were used. After a 6-month follow-up, analysis showed multiple contests with college students were a more suitable and effective strategy to raise the rate of smoking abstinence than a single contest, while there were no strong signals regarding the counselling.

## Materials and methods

### Data

#### Study population

The adult smokers who are at least 18 years reside in Pakistan and smoke cigarettes daily. Additionally, they are ready to meet the inclusion requirements and motivated to establish a quit date within the next 2 weeks of recruiting. Note that other nicotine- and non-nicotine-based cessation therapies will not be allowed during the trial.

#### Inclusion criteria

The potential participants for the trial will have to meet the following requirements.

- ◦ Participants are at least 18 years old. Upper age limit is 65 years.
- ◦ Smoke more than 10 combustible cigarettes a day at the time of study enrollment
- ◦ Smoking cigarettes for at least a year
- ◦ Participants are willing to stop combustible smoking.
- ◦ Participants are ready to sign a written consent form.
- ◦ There can only be one applicant per household.
- ◦ Own a phone that supports text messaging

#### Exclusion criteria

The potential participants will be not considered in case they are the following:

- Women who are pregnant
- Currently using other nicotine- and non-nicotine-based cessation therapies
- Females who intend to become pregnant during the trial’s participation term
- Experiencing chest pain or another cardiovascular event or procedure (e.g., heart attack, stroke, insertion of stent, bypass surgery).

#### Sampling strategy and sample selection

Keeping in view the study objectives, the process of deciding the number of observations/sample respondents would be made through the determination of sample size, which is based on the extent and intensity of variation and heterogeneity in the subject population. For a population with lower variation, a small sample is adequate and vice versa, ceteris paribus. In empirical studies aimed at representing the salient features of the population under study, the sample size is important. Before calculating the sample size, a few details about the target population, including its size, variance, margin of error, and desired level of confidence in empirical estimates of important variables, are required [56]. A major constraint in arriving at the ideal sample size is the lack of adequate information and data regarding the standard deviation of variables/indicators. In the absence of this specific information, this study referenced international studies conducted in a similar design, although some variations were noted. To prevent bias, the study opted for the most suitable sampling method considering the budget constraints and local limitations related to variations in the target population. The study employed the equivalence trial formula to estimate the primary outcome, which focused on reducing the use of combustible cigarettes through the provided interventions (Table 1).

$${n}_1={\left({Z}_{1-\frac{\alpha }{2}}+{Z}_{1-\beta}\right)}^2\ \frac{p_1\left(1-{p}_1\right)+{p}_2\left(1-{p}_2\right)}{{\left({p}_1-{p}_2\right)}^2}$$

where

$${p}_1= Proportion\ of\ outcome\ from\ group-1$$

$${p}_2= Proportion\ of\ outcome\ from\ group-2$$

$$\alpha = Level\ of\ significance$$

$$1-\beta = Power\ of\ test$$

$${Z}_{1-\alpha /2}=Z\ value\ of\ corresponding\ level\ of\ significance$$

$${Z}_{1-\beta }=Z\ value\ of\ corresponding\ level\ of\ power$$

$${n}_1= Sample\ size\ for\ one\ group$$

**Table 1:** Sample size calculation based on previous studies

Indicators	Information	Study: Hajek et al. (2019) [50]	Study: Smith et al. (2022) [12]	Study: Rigotti et al. (2018) [14]	Study: Caponnetto et al. (2013) [16]
	Rounded figures				
Proportion of outcome from group 1 (p1)	0.18	18%	26%	10%	14%
Proportion of outcome from group 2 (P2)	0.09	9%	6%	26%	5%
Level of significance (alpha)	0.05				
Power (1-beta)	0.8				
Z alpha	1.96				
Z beta	0.84				
Sample size group 1 (n1)	222.1	222	49	86	163

Based on the aforementioned formulas, from Hajek et al. [50], Smith et al. [12], Rigotti et al. [14], and Caponnetto et al. [16] derived 222, 49, 86, and 163 for single group respectively. According to acceptable norms and standards, the selected sample should represent the population. By the safe decision and following the abovementioned procedure, this study proposes a sample size of 600 participants, which will be randomly divided into three groups as follows:

- E-cigarette: The 200 participants will receive e-cigarettes along with basic care counselling.
- Nicotine pouches: The 200 participants will receive nicotine pouches along with basic care counselling.
- Basic care counselling: The 200 participants will receive basic care counselling.

Under the Lead Researcher Dr. Abdul Hameed, ARI team will conduct interview of participants and assign and unique ID to each potential participant at the screening stage. After that, the lead research Dr. A. H. will generate the allocation sequence and will assign participants to interventions by using STATA software for the randomization.

#### Recruitment strategies

This study will establish recruitment centers in two metropolitan districts: Islamabad and Rawalpindi. Participants will be enrolled based on their eligibility criteria through local mobilization. An ARI expert will conduct screening interviews. Through this independent and robust recruitment strategy, the study will achieve the required sample size. Face-to-face adherence reminder sessions will be held during the initial recruitment and at each subsequent study visit. These sessions will cover the following:

- Emphasizing the significance of adhering to study guidelines for the daily usage of products
- Providing instructions regarding the consumption of study products, such as proper dosage timing, storage guidelines, and the importance of taking the prescribed dose as a whole
- Follow-up sessions will take place during subsequent visits. Participants will be inquired about any difficulties they might be experiencing in adhering to their study interventions. These sessions will involve a brief discussion on the reasons behind missed doses and will offer simple strategies to enhance adherence.

### Study design

After the screening of the participants, this study will conduct a baseline survey to evaluate smoking status, smoking behavior, sociodemographic characteristics, health status, economic barriers, and motivation to stop smoking before the intervention and try to make a balance randomization between the control and the treatment groups. After completing the baseline survey, the three-arm study design would randomize 600 participants into three groups.

- E-cigarette: The 200 participants will receive e-cigarettes along with basic care counselling.
- Nicotine pouches: The 200 participants will receive nicotine pouches along with basic care counselling.
- Basic care counselling: The 200 participants will receive basic care counselling.

Four basic care counselling sessions will be held over the course of 48 weeks with the supply of e-cigarettes and nicotine pouches. These e-cigarettes and nicotine pouches will be provided in line with the indicated tastes and flavors. Every 12 weeks, a standard care counselling session will be offered, followed by a study visit to track any alterations in the user’s physical or mental health as well as any side effects from using nicotine pouches or e-cigarettes. All participants will complete a follow-up survey at 60 weeks. However, the provision of e-cigarettes and nicotine pouches will be stopped after the first 48 weeks.

**Fig. 1:**
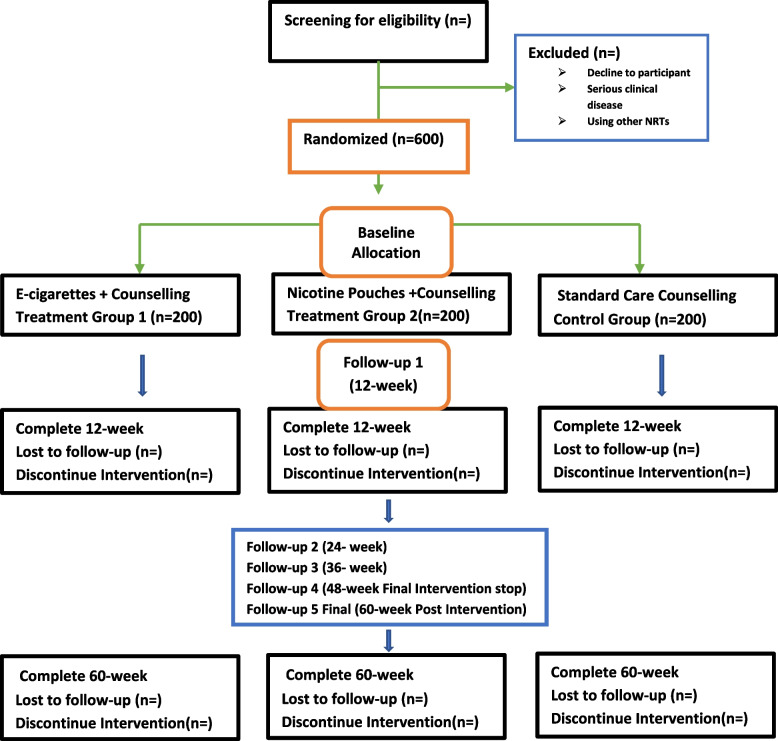
Consolidated Standards of Reporting Trial flow chart

**Fig. 2:**
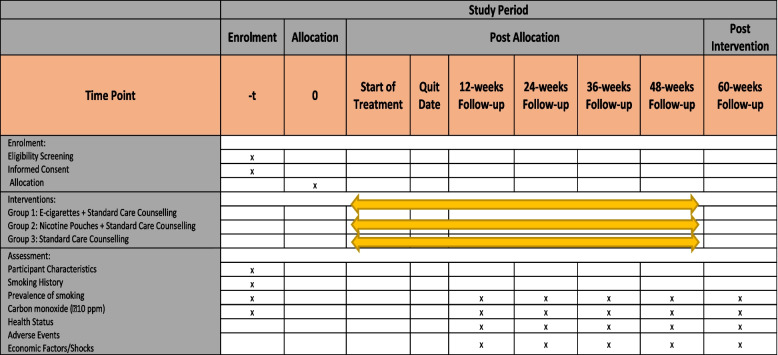
Participant study schedule and SPIRIT checklist

The remaining 12 weeks will be followed without providing any e-cigarettes or nicotine pouches. The participants must buy these items on their own. The overall five follow-ups will be conducted over the course of 60 weeks. Of these, four follow-ups will be during the period in which the participants received interventions, and one follow-up will be post-intervention period of 48 weeks. Additionally, a flowchart depicting the unified requirements of reporting trial is shown in Figs. 1 and 2 along with the explanation of the participants’ research schedule.

#### Primary outcomes

The primary outcome will be long-term change in health status. The best proxy indication for demonstrating a reduction in toxin intake from tobacco smoking that is adequate to result in a clinically relevant long-term health benefit is unknown [59]. In the absence of more accurate health indicators, the change in smoking rate from baseline and the smoking cessation will be the primary outcomes. This study will evaluate the impact of e-cigarettes and nicotine pouches on cigarettes per day (CPD and smoking cessation (7-day point abstinence) at weeks 24 and 48 (the study period ends in 60 weeks. At 48 weeks, ARI will be ending the provision of the interventions). Self-reported point-prevalence abstinence in the previous week with biochemical validation will be exhaled carbon monoxide less than 10 parts per million (PPM).

#### Secondary outcomes

Secondary outcomes will include 7-day point-prevalence abstinence (at all subsequent checkups; biochemically validated at weeks 12, 24, 36, 48, and 60). As a secondary goal, this study will analyze the harm-reduction effect of e-cigarettes and nicotine pouches, as well as the analysis of adverse events.

### Statistical analysis

To provide a summary position of the important variables of the population under clinical trial, a univariate analysis will be employed for data analysis and reporting. To examine the cross-relationship and association between selected variables, bivariate analysis and cross tabs will be used. Univariate data analysis is relevant and provides easy to understand information for focusing on a given variable at a time. The bivariate data analysis is helpful in examining the relationship and association between selected variables based on the understanding from previous studies or the theory. The differences in intervention indicators between the control and the treatment groups will be evaluated with the simple *t*-test and variance analysis.

The information obtained from the baseline survey will help in the planning (refine targeting, indicators to monitor), recommendations toward study design, implementation, and fine-tuning of the intervention objectives, backed by evidence-based research. However, the real execution of true experiment in the field is almost impossible due to heterogeneity of the beneficiaries. Therefore, we propose an alternate but similar approach, i.e., quasi-experimental difference in difference (DID) design, which is a close form of experimental research, widely used in the social sciences. The second-best option would be propensity score matching (PMS) technique to estimate the average treatment effect of the interventions.

The basic technique for taking DID for average and percentages of project is also referred to as the double difference evaluation design. It can measure change in outcome variables over time and across groups (beneficiaries compared with non-beneficiaries), a standard practice to ascertain and establish the attribution of a program in terms of outcome indicators. The sample requirements for a DID design are smaller than other impact evaluation designs. Table 2 and its accompanying chart illustrate how the DID estimator will measure the treatment effects of the program. In order to mitigate attrition bias, the analysis incorporates outcome data from all participants, irrespective of their adherence to the study protocol. This approach, known as “intention-to-treat” analysis, encompasses both randomized participants, serving as the recommended strategy for analysis. Additionally, the protocol must outline the intended approach for managing missing data during analysis. It should articulate the proposed methods for estimating missing outcome data, such as imputation, along with specifications regarding the variables employed in this process, if possible.

**Table 2:** The double difference design to measure treatment effect

	Baseline value of indicator	Follow-up value of indicator	Difference over time
Beneficiary group (intervention)	a	c	c–a
Non-beneficiary	B	d	d–b
(comparison)			
Difference over group	a–b	c–d	DID (treatment effect) = (d–b)–(c–a)
			or
			(a–b)–(c–d)

Depending upon the measurement scale of each indicator, the cells (a, b, c, and d) in the table can be primary and secondary outcomes indicators

### Data handling, monitoring, and recordkeeping

#### Survey instrument

In the clinical trial, structured questionnaires will gather data on respondent socioeconomic status, combustible smoking usage, intervention use, health status, and side effects of intervention products, etc. All essential topics will have clear definitions included in the questionnaire so that the interviewer can refer to them as and when needed. The questionnaire, translated into the local language of Urdu, will have instructional comments. Each question will be carefully reviewed to ensure elimination of leading or biased questions. On the Android-based census and survey processing system (CSpro) application, data will be gathered. Tablets using the Android operating system will be connected to a local server to guarantee security and data consistency. The program will allow users to save all collected data offline.

#### Content validity

Data from the field will be automatically recorded in the Android-based CSpro application. The pre-testing technique will check the internal consistency of questions and correct them in accordance with the local and clinical trial requirements.

#### Data collection plan: retention

This study will enroll only the potential candidates who will continue until the end of the trial. Additionally, this study will also enroll a higher number of participants than the optimum sample size to mitigate sample bias.

#### Management and quality control mechanism

To ensure authenticity and quality of data, a three-tier quality control mechanism will be followed in the field.

**Fig. 3:**
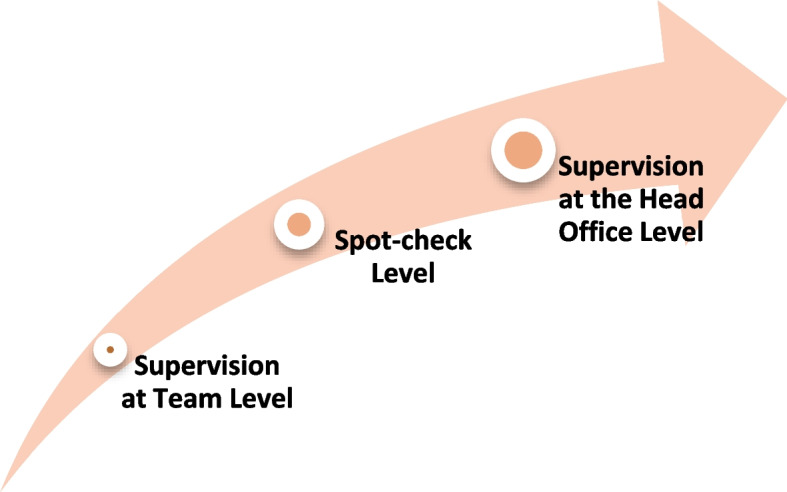
Three-tier management and quality control mechanism

- Tier 1—supervision at team level: The supervisors will ensure enumeration targets are met and keep an overall check on the field activities at the team level. Team leaders will ensure adherence to survey guidelines.
- Tier 2—spot-check level: Team from the head office will conduct surprise spot-checks/visits to ensure efficient management and control.
- Tier 3—supervision at the head office level: Head office staff comprising the core team will regularly monitor the survey/visit progress. The core team will also run consistency and reliability checks on the data received on a regular basis (Fig. 3).

The Android version of KoBo tool will be employed for data collection. This software is highly comprehensive and includes data validity checks. The gathered data will be transformed into a locally centralized survey under the supervision of the research lead. Following the collection of field data, real-time analysis concerning data discrepancies and quality will be conducted using a predefined STATA Do file. This file will encompass data values, ranges, and other quality checks.

#### Data cleaning and coding

Capitalizing on our experience in conducting and managing field surveys, we will develop a data coding strategy to ensure the following:

- ◦ Each respondent/interviewee selected will be assigned a unique identifier.
- ◦ Each enumerator and supervisor will have a unique code that will be recorded on all questionnaires completed by a given team. This will ensure the quality assurance of data management by evaluating the performance of everyone separately.
- ◦ All geographical denominations will have a unique location code in the database.
- ◦ Assign variable names, values, and labels to clean the database.

## Discussion

Pakistan lacks adequate number of programs to help smokers quit smoking or reduce their use of tobacco. This is especially the case for low- and middle-income communities where most smokers use combustible, cheap cigarettes. The smokers in these communities need assistance in order to quit or reduce combustible smoking. The tobacco control efforts in Pakistan are unfortunately ignoring the role of tobacco harm reduction products in reducing the prevalence of smoking. This may be because of the lack of evidence on the possible role of THR in assisting the smokers quit or reduce harm to their health.

Lack of knowledge about THR and their higher prices in Pakistan are a barrier to adopting them. People used THR items on their own, including e-cigarettes, nicotine pouches, and non-nicotine replacement therapies (non-NRTs).

E-cigarettes and other THR products are imported legally as a consumer good and are taxable. Currently, the use of THR, mainly e-cigarettes, is unregulated and limited to the upper and middle classes due to their higher prices and limited access to the economically deprived areas of Pakistan. Though the organizations working on the tobacco control are demanding a ban on the THR products in Pakistan, their use continues in a regulatory vacuum. The lack of sufficient and evidence-based data-related THR and its application in Pakistan is a challenge. Evidence-based studies on e-cigarettes and other nicotine products may help Pakistan in formulating effective policies to reduce the harm caused by combustible smoking. This study results will be used to inform the governments of Pakistan, decision-makers, and researchers about the effects of using e-cigarettes and nicotine pouches vis-à-vis quitting smoking or switching to lesser harmful alternatives. This will help to define the role of e-cigarettes and other alternative nicotine delivery systems as a potential smoking cessation aid.

### Supplementary Information

**Additional file 1.** SPIRIT Checklist for Trials.

**Additional file 2.**

## Data Availability

All relevant data can be provided on demand.
