# Supplementary material for: Clinical study protocol on electronic cigarettes and nicotine pouches for smoking cessation in Pakistan: a randomized controlled trial
Source: Trials. 2024 Jan 2;25:9. doi: 10.1186/s13063-023-07876-y (PMC10759381; doi:10.1186/s13063-023-07876-y)
Supplement: Supplementary file 1 — Additional file 1. SPIRIT Checklist for Trials. [file 13063_2023_7876_MOESM1_ESM.docx]

# **SPIRIT Checklist for *Trials***

Complete this checklist by entering the page and line numbers where each of the items listed below can be found in your manuscript.

Your manuscript may not currently address all the items on the checklist. Please modify your text to include the missing information. If you are certain that an item does not apply, please state "n/a" and provide a short explanation. **Leaving an item blank or stating “n/a” without an explanation will lead to your manuscript being returned before review.**

Upload your completed checklist as an additional file when you submit to *Trials*. You must reference this additional file in the main text of your protocol submission. The completed SPIRIT figure must be included within the main body of the protocol text and can be downloaded here: <http://www.spirit-statement.org/schedule-of-enrolment-interventions-and-assessments/>

In your methods section, please state that you used the SPIRIT reporting guidelines, and cite them as:

Chan A-W, Tetzlaff JM, Gøtzsche PC, Altman DG, Mann H, Berlin J, Dickersin K, Hróbjartsson A, Schulz KF, Parulekar WR, Krleža-Jerić K, Laupacis A, Moher D. SPIRIT 2013 Explanation and Elaboration: Guidance for protocols of clinical trials. BMJ. 2013;346:e7586

|  |  | **Reporting Item** | **Page and Line Number** | **Reason if not applicable** |
| --- | --- | --- | --- | --- |
| **Administrative information** | | | | |
| Title | [#1](https://www.goodreports.org/reporting-checklists/spirit/info/#1) | Descriptive title identifying the study design, population, interventions, and, if applicable, trial acronym | Clinical Study Protocol on Electronic Cigarettes and Nicotine Pouches for Smoking Cessation in Pakistan: A Randomized Controlled Trial |  |
| Trial registration | [#2a](https://www.goodreports.org/reporting-checklists/spirit/info/#2a) | Trial identifier and registry name. If not yet registered, name of intended registry | U.S. National Institutes of Health Clinical Trials for clinical study protocol registration: https://clinicaltrials.gov/ct2/show/NCT05715164 |  |
| Trial registration: data set | [#2b](https://www.goodreports.org/reporting-checklists/spirit/info/#2b) | All items from the World Health Organization Trial Registration Data Set | See S1 |  |
| Protocol version | [#3](https://www.goodreports.org/reporting-checklists/spirit/info/#3) | Date and version identifier | Protocol version 1.0, 14-12-2022 |  |
| Funding | [#4](https://www.goodreports.org/reporting-checklists/spirit/info/#4) | Sources and types of financial, material, and other support | This study was funded by a grant from the Foundation for a Smoke-Free World, a US nonprofit 501(c)(3) private foundation with a mission to end smoking in this generation. The foundation accepts charitable gifts from the PMI Global Services Inc. (PMI). Under the Foundation’s Bylaws and Pledge Agreement with PMI, the foundation is independent from PMI and the tobacco industry. |  |
| Roles and responsibilities: contributorship | [#5a](https://www.goodreports.org/reporting-checklists/spirit/info/#5a) | Names, affiliations, and roles of protocol contributors | Dr Abdul Hameed is the principal investigator and senior researcher who has drafted the protocol, study design, sampling, preparation of data tools and analysis. He has critically reviewed and revised the protocol. Daud Malik helped to critically review and revise the protocol. All authors read and approved the final protocol for publication |  |
| Roles and responsibilities: sponsor contact information | [#5b](https://www.goodreports.org/reporting-checklists/spirit/info/#5b) | Name and contact information for the trial sponsor | Foundation for a Smoke-Free World, a US |  |
| Roles and responsibilities: sponsor and funder | [#5c](https://www.goodreports.org/reporting-checklists/spirit/info/#5c) | Role of study sponsor and funders, if any, in study design; collection, management, analysis, and interpretation of data; writing of the report; and the decision to submit the report for publication, including whether they will have ultimate authority over any of these activities | This study was funded by a grant from the Foundation for a Smoke-Free World, a US nonprofit 501(c)(3) private foundation with a mission to end smoking in this generation. ARI's research and perspectives are shaped solely by its own research processes and intellectual exploration, with financial support from a foundation serving as a necessary but non-influential component of its operations. |  |
| Roles and responsibilities: committees | [#5d](https://www.goodreports.org/reporting-checklists/spirit/info/#5d) | Composition, roles, and responsibilities of the coordinating centre, steering committee, endpoint adjudication committee, data management team, and other individuals or groups overseeing the trial, if applicable (see Item 21a for data monitoring committee) | N/A | This study used Tier 1-Supervision at Team Level and Tier 2–Spot-check Level: Team from the head office will conduct surprise spot-checks /visits to ensure efficient management and control, and Tier 3–Supervision at the Head Office Level: Head office staff comprising the core team will regularly monitor the survey/visit progress. |
| **Introduction** |  |  | 2,38 |  |
| Background and rationale | [#6a](https://www.goodreports.org/reporting-checklists/spirit/info/#6a) | Description of research question and justification for undertaking the trial, including summary of relevant studies (published and unpublished) examining benefits and harms for each intervention | There are more than a billion consumers of higher risk tobacco products worldwide, including cigarettes, bidis, and cigars. The World Health Organization (WHO) estimates the tobacco pandemic kills more than eight million people annually [1]. In LMICs, where the burden of smoking-related illness and mortality is highest, the majority of the world's 1.1 billion smokers reside [2]. With 29 million active adult tobacco users, Pakistan is one of the most vulnerable LMICs in South Asia. More than 45% households in Pakistan use tobacco [3].  Estimates say tobacco kills around 163,600 people each year in Pakistan. Secondhand smoke is responsible for over 31,000 of these deaths [4]. Annually on average 82 billion cigarettes are consumed [5]. A total of 16.8 million adults who work indoors and 56.3 million at home are exposed to secondhand smoking [6]. The total cost of all smoking-related illnesses and fatalities in 2019 was Rs615.07 billion ($3.85 billion). However, the Rs120 billion collected in taxes from the tobacco industry in 2019 covered only 20% of the overall costs associated with smoking [7].  Pakistan’s tobacco control initiatives have focused on restricting or disrupting the demand for cigarettes, with little emphasis on cessation. The country has employed various initiatives, such as cessation clinics, public awareness campaigns, and restrictions. However, the number of smokers has grown over time. The efficacy of such therapies is questionable and requires factual research. Furthermore, information about cigarette cessation services is not readily available; few people are aware of this. Even well-educated young smokers who want to quit are unaware of the cessation programs [8]. Due to a lack of knowledge about cessation services, almost half of all tobacco cessation attempts in Pakistan are unassisted [9].  Recent studies on barriers to smoking cessation suggest that the social interaction and friendships at work, home, and in public are the primary motivators for initiating smoking. The main deterrent to obtaining medical help to stop smoking seems to be lack of knowledge about its availability. The knowledge regarding THR, particularly e-cigarettes, can be described as imprecise. In Pakistan, tobacco control initiatives lack focus on quitting smoking. To help people stop smoking, medical and professional support is a must. This support should be provided with education campaigns about the harms of combustible smoking and the role of THR in smoking cessation [10-11]. On the other hand, both the public and health professionals have little knowledge regarding nicotine. More than two-thirds of doctors (70%) in Pakistan think nicotine causes cancer. With the use of research-based therapies and communication strategies, misconceptions about nicotine can be corrected |  |
| Background and rationale: choice of comparators | [#6b](https://www.goodreports.org/reporting-checklists/spirit/info/#6b) | Explanation for choice of comparators | THR is barely making its presence felt in Pakistan. Individuals and business owners of the THR products remain wary of any possible regulations/rules that may delay or shut down their businesses. There are no clear or defined rules and regulations governing the use of THR, including import, manufacturing, or product content. E-cigarettes and other THR products are legally imported as consumer goods and subject to taxation. The availability of data regarding THR products, and their use is limited. According to some estimates, the number of THR users in Pakistan is somewhere between 30,000 and 50,000 with unreliable evidence of dual use of conventional smoking and vaping. Most of the vaping outlets in Pakistan are in the upscale localities of major cities such as Karachi, Lahore, Rawalpindi, and Islamabad. |  |
| Objectives | [#7](https://www.goodreports.org/reporting-checklists/spirit/info/#7) | Specific objectives or hypotheses | In this context, this study uses a randomized controlled trial and primary data from two metropolitan districts – Islamabad and Rawalpindi – to examine the effectiveness and role of electronic cigarettes and nicotine pouches for smoking cessation in Pakistan. This would be the first nationwide clinical investigation into the effectiveness of nicotine pouches and e-cigarettes |  |
| Trial design | [#8](https://www.goodreports.org/reporting-checklists/spirit/info/#8) | Description of trial design including type of trial (eg, parallel group, crossover, factorial, single group), allocation ratio, and framework (eg, superiority, equivalence, non-inferiority, exploratory) | Allocation: Randomized  Interventional Model: Parallel Assignment  Interventional Model Description: The baseline survey will help to evaluate the smoking status, smoking behavior, socio-demographic characteristics, health status, etc. The three-arm study design would randomize 600 participants into three groups. Of the 600 participants, 200 each will receive e-cigarettes, nicotine pouches, and basic care counseling respectively. Four basic care counseling sessions will be held over the course of 48 weeks. Every 12 weeks, a standard care counseling session will be offered, followed by a study visit to track any alterations in the user's physical or mental health as well as any side effects from using nicotine pouches or e-cigarettes. All participants will complete a follow-up survey at 60 weeks. The provision of e-cigarettes and nicotine pouches will be stopped after the first 48 weeks. The remaining 12 weeks will be without the provision of any intervention and the participants must buy these items on their own.  Participant Group/Arm Intervention/Treatment  Experimental: E-cigarettes device plus liquid  The 200 participants will receive e-cigarettes along with basic care counseling for 48 weeks.  Device: E-cigarettes device plus liquid  The four basic care counseling sessions will be held over the course of 48 weeks with the supply of e-cigarettes and liquid. Every 12 weeks, a standard care counseling session will be offered, and it will be followed by a study visit to track any alterations in the user's physical or mental health as well as any side effects from using e-cigarettes.  Experimental: Nicotine Pouches  The 200 participants will receive nicotine pouches along with basic care counseling for 48 weeks.  Drug: Nicotine Pouches  The four basic care counseling sessions will be held over the course of 48 weeks with a supply of nicotine pouches. Every 12 weeks, a standard care counseling session will be offered, and it will be followed by a study visit to track any alterations in the user's physical or mental health as well as any side effects from using nicotine pouches.  Active Comparator: Basic care counseling about smoking cessation  The 200 participants will receive basic care counseling for 48 weeks.  Other: Basic care counseling about smoking cessation  The four basic care counseling sessions will be held over the course of 48 weeks. Every 12 weeks, a standard care counseling session will be offered, and it will be followed by a study visit to track any alterations in the user's physical or mental health. |  |
| **Methods: Participants, interventions, and outcomes** | | | | |
| Study setting | [#9](https://www.goodreports.org/reporting-checklists/spirit/info/#9) | Description of study settings (eg, community clinic, academic hospital) and list of countries where data will be collected. Reference to where list of study sites can be obtained | The adult smokers who are at least 18 years, reside in Pakistan, and smoke cigarettes daily. Additionally, they are ready to meet the inclusion requirements and motivated to establish a quit date within the next two weeks of recruiting. |  |
| Eligibility criteria | [#10](https://www.goodreports.org/reporting-checklists/spirit/info/#10) | Inclusion and exclusion criteria for participants. If applicable, eligibility criteria for study centres and individuals who will perform the interventions (eg, surgeons, psychotherapists) | The potential participants for the trial will have to meet the following requirements.  o Participants are at least 18 years old. Upper age limit is 65 years.  o Smoke more than 10 combustible cigarettes a day at the time of study enrollment.  o Smoking cigarettes for at least a year.  o Participants are willing to stop combustible smoking.  o Participants are ready to sign a written consent form.  o There can only be one applicant per household.  o Own a phone that supports text massaging. |  |
| Interventions: description | [#11a](https://www.goodreports.org/reporting-checklists/spirit/info/#11a) | Interventions for each group with sufficient detail to allow replication, including how and when they will be administered | After the screening of the participants, this study will conduct a baseline survey to evaluate smoking status, smoking behavior, socio-demographic characteristics, health status, economic barriers, and motivation to stop smoking before the intervention and try to make a balance randomization between the control and the treatment groups. After completing the baseline survey, the three-arm study design would randomize 600 participants into three groups.  o E-cigarette: The 200 participants will receive e-cigarettes along with basic care counselling.  o Nicotine Pouches: The 200 participants will receive nicotine pouches along with basic care counselling.  o Basic Care Counselling: The 200 participants will receive basic care counselling.  Four basic care counselling sessions will be held over the course of 48 weeks with the supply of e-cigarettes and nicotine pouches. These e-cigarettes and nicotine pouches will be provided in line with the indicated tastes and flavors. Every 12 weeks, a standard care counselling session will be offered, followed by a study visit to track any alterations in the user's physical or mental health as well as any side effects from using nicotine pouches or e-cigarettes. All participants will complete a follow-up survey at 60 weeks. However, the provision of e-cigarettes and nicotine pouches will be stopped after the first 48 weeks.  The remaining 12 weeks will be followed without providing any e-cigarettes or nicotine pouches. The participants must buy these items on their own. The overall five follow-ups will be conducted over the course of 60 weeks. Of these, four follow-ups will be during the period in which the participants received interventions and one follow-up will be post-intervention period of 48 weeks. Additionally, a flowchart depicting the unified requirements of reporting trial is shown in Figures 1 and 2 along with the explanation of the participants' research schedule. |  |
| Interventions: modifications | [#11b](https://www.goodreports.org/reporting-checklists/spirit/info/#11b) | Criteria for discontinuing or modifying allocated interventions for a given trial participant (eg, drug dose change in response to harms, participant request, or improving / worsening disease) | N/A | There is no needs of modification |
| Interventions: adherance | [#11c](https://www.goodreports.org/reporting-checklists/spirit/info/#11c) | Strategies to improve adherence to intervention protocols, and any procedures for monitoring adherence (eg, drug tablet return; laboratory tests) | Face-to-face adherence reminder sessions will occur during the initial recruitment and at each subsequent study visit. These sessions will cover the following:  Emphasizing the significance of adhering to study guidelines for the daily usage of products.  Providing instructions regarding the consumption of study products, such as proper dosage timing, storage guidelines, and the importance of taking the prescribed dose as a whole.  Follow-up sessions will take place during subsequent visits. Participants will be inquired about any difficulties they might be experiencing in adhering to their study interventions. These sessions will involve a brief discussion on the reasons behind missed doses and will offer simple strategies to enhance adherence. |  |
| Interventions: concomitant care | [#11d](https://www.goodreports.org/reporting-checklists/spirit/info/#11d) | Relevant concomitant care and interventions that are permitted or prohibited during the trial | The potential participants will be not considered in case they are:  o Women who are pregnant.  o Currently using other nicotine- and non-nicotine-based cessation therapies.  o Females who intend to become pregnant during the trial's participation term.  o Experiencing chest pain, or another cardiovascular event or procedure (e.g., heart attack, stroke, insertion of stent, bypass surgery).  Noted: The other nicotine- and non-nicotine-based cessation therapies will be not allowed during the trial |  |
| Outcomes | [#12](https://www.goodreports.org/reporting-checklists/spirit/info/#12) | Primary, secondary, and other outcomes, including the specific measurement variable (eg, systolic blood pressure), analysis metric (eg, change from baseline, final value, time to event), method of aggregation (eg, median, proportion), and time point for each outcome. Explanation of the clinical relevance of chosen efficacy and harm outcomes is strongly recommended | The primary outcome will be long-term change in health status. The best proxy indication for demonstrating a reduction in toxin intake from tobacco smoking that is adequate to result in a clinically relevant long-term health benefit is unknown [59]. In the absence of more accurate health indicators, the change in smoking rate from baseline and the smoking cessation will be the primary outcomes. This study will evaluate the impact of e-cigarettes and nicotine pouches on cigarettes per day (CPD and smoking cessation (Seven Day Point Abstinence) at weeks 24 and 48 (The study period ends in 60 weeks. At 48 weeks, ARI will be ending the provision of the interventions). Self-reported point-prevalence abstinence in the previous week with biochemical validation will be exhaled carbon monoxide less than 10 parts per million (PPM). |  |
| Participant timeline | [#13](https://www.goodreports.org/reporting-checklists/spirit/info/#13) | Time schedule of enrolment, interventions (including any run-ins and washouts), assessments, and visits for participants. A schematic diagram is highly recommended (see Figure) | Secondary outcomes will include seven-day point-prevalence abstinence (at all subsequent check-ups; biochemically validated at weeks 12, 24, 36, 48, and 60). As a secondary goal, this study will analyze the harm-reduction effect of e-cigarettes and nicotine pouches, as well as the analysis of adverse events. |  |
| Sample size | [#14](https://www.goodreports.org/reporting-checklists/spirit/info/#14) | Estimated number of participants needed to achieve study objectives and how it was determined, including clinical and statistical assumptions supporting any sample size calculations | Keeping in view the study objectives, the process of deciding the number of observations/sample respondents would be made through the determination of sample size, which is based on the extent and intensity of variation and heterogeneity in the subject population. For a population with lower variation, a small sample is adequate and vice versa, ceteris paribus. In empirical studies aimed at representing the salient features of the population under study, the sample size is important. Before calculating the sample size, a few details about the target population, including its size, variance, margin of error and desired level of confidence in empirical estimates of important variables are required [57]. A major constraint in arriving at the ideal sample size is the lack of adequate information and data regarding the standard deviation of variables /indicators. In the absence of this specific information, this study referenced international studies conducted in a similar design, although some variations were noted. To prevent bias, the study opted for the most suitable sampling method considering the budget constraints and local limitations related to variations in the target population. The study employed the equivalence trial formula to estimate the primary outcome, which focused on reducing the use of combustible cigarettes through the provided interventions.  $n_{1}=\left( Z_{1-\frac{\alpha}{2}}+Z_{1-\beta} \right)^{2} \frac{p_{1}\left( 1-p_{1} \right)+ p_{2}\left( 1-p_{2} \right)}{\left( p_{1}-p_{2} \right)^{2}}$  Where:   - $p_{1}=Proportion of outcome from group-1$ - $p_{2}=Proportion of outcome from group-2$ - $\alpha=Level of significance$ - $1-\beta=Power of test$ - $Z_{1-\alpha/2}=Z value of corresponding level of significance$ - $Z_{1-\beta}=Z value of corresponding level of power$ - $n_{1}=Sample size for one group$   **Table 1: Sample size calculation on the based of previous studies**   \| Indicators \| Information \| Study: Hajek et al. 2019 \| Study: Smith et al. 2022 \| Study: Rigotti et al. 2018 \| Study: Caponnetto et al. 2013 \| \| --- \| --- \| --- \| --- \| --- \| --- \| \|  \| Rounded Figures \| \| \| \| \| \| Proportion of outcome from group-1 (p1) \| 0.18 \| 18% \| 26% \| 10% \| 14% \| \| Proportion of outcome from group-2 (P2) \| 0.09 \| 9% \| 6% \| 26% \| 5% \| \| Level of Significance (alpha) \| 0.05 \|  \| \| \| \| \| Power (1-beta) \| 0.8 \| \| Z Alpha \| 1.96 \| \| Z beta \| 0.84 \| \| Sample size Group 1 (n1) \| 222.1 \| 222 \| 49 \| 86 \| 163 \|   Based on the aforementioned formulas, from Hajek et al. (2019), Smith et al. (2022), Rigotti et al. (2018) and Caponnetto et al. (2013) derived 222, 49, 86 and 163 for single group respectively. According to acceptable norms and standards, the selected sample should represent the population. by the safe decision and following the above-mentioned procedure, this study proposes a sample size of 600 participants, which will be randomly divided into three groups as:   - - E-cigarette: The 200 participants will receive e-cigarettes along with basic care counselling.   - Nicotine Pouches: The 200 participants will receive nicotine pouches along with basic care counselling.   - Basic Care Counselling: The 200 participants will receive basic care counselling. |  |
| Recruitment | [#15](https://www.goodreports.org/reporting-checklists/spirit/info/#15) | Strategies for achieving adequate participant enrolment to reach target sample size | This study will establish recruitment centers in two metropolitan districts: Islamabad and Rawalpindi. Participants will be enrolled based on their eligibility criteria through local mobilization. An ARI expert will conduct screening interviews. Through this independent and robust recruitment strategy, the study will achieve the required sample size. |  |
| **Methods: Assignment of interventions (for controlled trials)** | | | | |
| Allocation: sequence generation | [#16a](https://www.goodreports.org/reporting-checklists/spirit/info/#16a) | Method of generating the allocation sequence (eg, computer-generated random numbers), and list of any factors for stratification. To reduce predictability of a random sequence, details of any planned restriction (eg, blocking) should be provided in a separate document that is unavailable to those who enrol participants or assign interventions | After the screening of the participants, this study will conduct a baseline survey to evaluate smoking status, smoking behavior, socio-demographic characteristics, health status, economic barriers, and motivation to stop smoking before the intervention and try to make a balance randomization between the control and the treatment groups. After completing the baseline survey, the three-arm study design would randomize 600 participants into three groups. For this, computer-generated random (STATA Randomized Sampling) program will be used to select and assigned the intervention. |  |
| Allocation concealment mechanism | [#16b](https://www.goodreports.org/reporting-checklists/spirit/info/#16b) | Mechanism of implementing the allocation sequence (eg, central telephone; sequentially numbered, opaque, sealed envelopes), describing any steps to conceal the sequence until interventions are assigned | This study will establish recruitment centers in two metropolitan districts: Islamabad and Rawalpindi. Participants will be enrolled based on their eligibility criteria through local mobilization. An ARI expert will conduct screening interviews. Through this independent and robust recruitment strategy, the study will achieve the required sample size. To accomplish this, a computer-generated randomization program (STATA Randomized Sampling) will be utilized to select and assign the intervention. |  |
| Allocation: implementation | [#16c](https://www.goodreports.org/reporting-checklists/spirit/info/#16c) | Who will generate the allocation sequence, who will enrol participants, and who will assign participants to interventions | Under the Lead Researcher Dr. Abdul Hameed, ARI team will conduct interview of participants and assign and unique ID to each potential participant at the screening stage. After that, the lead research Dr. AH will generate the allocation sequence and will assign participants to interventions by using STATA software for the randomization. |  |
| Blinding (masking) | [#17a](https://www.goodreports.org/reporting-checklists/spirit/info/#17a) | Who will be blinded after assignment to interventions (eg, trial participants, care providers, outcome assessors, data analysts), and how | There is no need of blinding |  |
| Blinding (masking): emergency unblinding | [#17b](https://www.goodreports.org/reporting-checklists/spirit/info/#17b) | If blinded, circumstances under which unblinding is permissible, and procedure for revealing a participant’s allocated intervention during the trial |  | There is no need of blinding |
| **Methods: Data collection, management, and analysis** | | | | |
| Data collection plan | [#18a](https://www.goodreports.org/reporting-checklists/spirit/info/#18a) | Plans for assessment and collection of outcome, baseline, and other trial data, including any related processes to promote data quality (eg, duplicate measurements, training of assessors) and a description of study instruments (eg, questionnaires, laboratory tests) along with their reliability and validity, if known. Reference to where data collection forms can be found, if not in the protocol | In the clinical trial, structured questionnaires will gather data on respondent socioeconomic status, combustible smoking usage, intervention use, health status, and side effects of intervention products, etc. All essential topics will have clear definitions included in the questionnaire so that the interviewer can refer to them as and when needed. The questionnaire, translated into the local language of Urdu, will have instructional comments. Each question will be carefully reviewed to ensure elimination of leading or biased questions. On the Android-Based Census and Survey Processing System (CSpro) application, data will be gathered. Tablets using the Android operating system will be connected to a local server to guarantee security and data consistency. The program will allow users to save all collected data offline |  |
| Data collection plan: retention | [#18b](https://www.goodreports.org/reporting-checklists/spirit/info/#18b) | Plans to promote participant retention and complete follow-up, including list of any outcome data to be collected for participants who discontinue or deviate from intervention protocols | This study will enroll only the potential candidates who will continue until the end of the trial. Additionally, this study will also enroll a higher number of participants than the optimum sample size to mitigate sample bias. |  |
| Data management | [#19](https://www.goodreports.org/reporting-checklists/spirit/info/#19) | Plans for data entry, coding, security, and storage, including any related processes to promote data quality (eg, double data entry; range checks for data values). Reference to where details of data management procedures can be found, if not in the protocol | To ensure authenticity and quality of data, a three-tier quality control mechanism will be followed in the field.  • Tier 1-Supervision at Team Level: The supervisors will ensure enumeration targets are met and keep an overall check on the field activities at the team level. Team leaders will ensure adherence to survey guidelines.  • Tier 2–Spot-check Level: Team from the head office will conduct surprise spot-checks /visits to ensure efficient management and control.  • Tier 3–Supervision at the Head Office Level: Head office staff comprising the core team will regularly monitor the survey/visit progress. The core team will also run consistency and reliability checks on the data received on a regular basis.  The Android version of KoBo tool will be employed for data collection. This software is highly comprehensive and includes data validity checks. The gathered data will be transformed into a locally centralized survey under the supervision of the research lead. Following the collection of field data, real-time analysis concerning data discrepancies and quality will be conducted using a predefined STATA Do file. This file will encompass data values, ranges, and other quality checks. |  |
| Statistics: outcomes | [#20a](https://www.goodreports.org/reporting-checklists/spirit/info/#20a) | Statistical methods for analysing primary and secondary outcomes. Reference to where other details of the statistical analysis plan can be found, if not in the protocol | To provide a summary position of the important variables of the population under clinical trial, a univariate analysis will be employed for data analysis and reporting. To examine the cross-relationship and association between selected variables, bivariate analysis and cross tabs will be used. Univariate data analysis is relevant and provides easy to understand information for focusing on a given variable at a time. The bivariate data analysis is helpful in examining the relationship and association between selected variables based on the understanding from previous studies or the theory. The differences in intervention indicators between the control and the treatment groups will be evaluated with the simple t-test and variance analysis. Furthermore, this study will utilize RCT regression analysis incorporating covariates. The study also will conduct quasi-experimental analysis due to the heterogeneity among beneficiaries. The study results will be compared using both pure and quasi-experimental designs. In developing countries, pure randomization is challenging to implement in the field, leading to potential bias in population-based results. Therefore, we will employ quasi-experimental techniques like DID (Difference-in-Differences) and PSM (Propensity Score Matching) to mitigate this issue. |  |
| Statistics: additional analyses | [#20b](https://www.goodreports.org/reporting-checklists/spirit/info/#20b) | Methods for any additional analyses (eg, subgroup and adjusted analyses) | The information obtained from the baseline survey will help in the planning (refine targeting, indicators to monitor), recommendations toward study design, implementation and fine-tuning of the intervention objectives, backed by evidence-based research. According to the primary and secondary outcomes (Number of participants reported adverse events of e-cigarettes or nicotine pouches, change in the number of combustible cigarettes per day and point-prevalence abstinence), this study will use RCT regression analysis along with covariates. However, in the real execution of true experiment in the field is almost impossible due to heterogeneity of the beneficiaries. Therefore, we propose an alternate but similar approach i.e., quasi-experimental difference in difference (DID) design, which is a close form of experimental research, widely used in the social sciences. The second-best option would be Propensity Score Matching (PMS) technique to estimate the average treatment effect of the interventions and matching respondent socioeconomic factors. These methods will compare with the pure RCT regression analysis to understand the actual essence of the intervention. |  |
| Statistics: analysis population and missing data | [#20c](https://www.goodreports.org/reporting-checklists/spirit/info/#20c) | Definition of analysis population relating to protocol non-adherence (eg, as randomised analysis), and any statistical methods to handle missing data (eg, multiple imputation) | In order to mitigate attrition bias, the analysis incorporates outcome data from all participants, irrespective of their adherence to the study protocol. This approach, known as "intention to treat" analysis, encompasses both randomized participants, serving as the recommended strategy for analysis. Additionally, the protocol must outline the intended approach for managing missing data during analysis. It should articulate the proposed methods for estimating missing outcome data, such as imputation, along with specifications regarding the variables employed in this process, if possible. |  |
| **Methods: Monitoring** | | | | |
| Data monitoring: formal committee | [#21a](https://www.goodreports.org/reporting-checklists/spirit/info/#21a) | Composition of data monitoring committee (DMC); summary of its role and reporting structure; statement of whether it is independent from the sponsor and competing interests; and reference to where further details about its charter can be found, if not in the protocol. Alternatively, an explanation of why a DMC is not needed | While there isn't a specific Data Monitoring Committee (DMC) in place, the proficient teams supervised by the Research Lead will handle all aspects of data collection and management.  However, to ensure authenticity and quality of data, a three-tier quality control mechanism will be followed in the field.  • Tier 1-Supervision at Team Level: The supervisors will ensure enumeration targets are met and keep an overall check on the field activities at the team level. Team leaders will ensure adherence to survey guidelines.  • Tier 2–Spot-check Level: Team from the head office will conduct surprise spot-checks /visits to ensure efficient management and control.  • Tier 3–Supervision at the Head Office Level: Head office staff comprising the core team will regularly monitor the survey/visit progress. The core team will also run consistency and reliability checks on the data received on a regular basis. |  |
| Data monitoring: interim analysis | [#21b](https://www.goodreports.org/reporting-checklists/spirit/info/#21b) | Description of any interim analyses and stopping guidelines, including who will have access to these interim results and make the final decision to terminate the trial | N/A | Interim analyses are typically conducted in trials with a Data Monitoring Committee (DMC), involving longer recruitment durations and potentially significant outcomes. However, this study focuses on a shorter trial examining smoking cessation and the behavior of smokers regarding the cessation aid. |
| Harms | [#22](https://www.goodreports.org/reporting-checklists/spirit/info/#22) | Plans for collecting, assessing, reporting, and managing solicited and spontaneously reported adverse events and other unintended effects of trial interventions or trial conduct | N/A | There are no anticipated harms |
| Auditing | [#23](https://www.goodreports.org/reporting-checklists/spirit/info/#23) | Frequency and procedures for auditing trial conduct, if any, and whether the process will be independent from investigators and the sponsor | N/A | Frequency and procedures for auditing trial is not required. |
| **Ethics and dissemination** | | | | |
| Research ethics approval | [#24](https://www.goodreports.org/reporting-checklists/spirit/info/#24) | Plans for seeking research ethics committee / institutional review board (REC / IRB) approval | A clearance from the ARI board and the consent of the participants will be obtained before recruiting them for the study (see Additional documents, “Approval Letter” and “Clinical Protocol Approved by R&DC”. |  |
| Protocol amendments | [#25](https://www.goodreports.org/reporting-checklists/spirit/info/#25) | Plans for communicating important protocol modifications (eg, changes to eligibility criteria, outcomes, analyses) to relevant parties (eg, investigators, REC / IRBs, trial participants, trial registries, journals, regulators) | N/A | Protocol approved by the ARI ethic and research committee |
| Consent or assent | [#26a](https://www.goodreports.org/reporting-checklists/spirit/info/#26a) | Who will obtain informed consent or assent from potential trial participants or authorised surrogates, and how (see Item 32) | ARI will take Consent |  |
| Consent or assent: ancillary studies | [#26b](https://www.goodreports.org/reporting-checklists/spirit/info/#26b) | Additional consent provisions for collection and use of participant data and biological specimens in ancillary studies, if applicable | N/A | There is no required any additional consent |
| Confidentiality | [#27](https://www.goodreports.org/reporting-checklists/spirit/info/#27) | How personal information about potential and enrolled participants will be collected, shared, and maintained in order to protect confidentiality before, during, and after the trial | A clearance from the ARI board and the consent of the participants will be obtained before recruiting them for the study (see Additional documents, “Approval Letter” and “Clinical Protocol Approved by R&DC”. The personal information of the participant will not share with any one and it will be confidential. |  |
| Declaration of interests | [#28](https://www.goodreports.org/reporting-checklists/spirit/info/#28) | Financial and other competing interests for principal investigators for the overall trial and each study site | The authors declare that they have no competing interests. |  |
| Data access | [#29](https://www.goodreports.org/reporting-checklists/spirit/info/#29) | Statement of who will have access to the final trial dataset, and disclosure of contractual agreements that limit such access for investigators | The final trial dataset will be accessible to the public on both the clinicaltrials.gov and ARI website. |  |
| Ancillary and post trial care | [#30](https://www.goodreports.org/reporting-checklists/spirit/info/#30) | Provisions, if any, for ancillary and post-trial care, and for compensation to those who suffer harm from trial participation | N/A | The trail is highly straightforward, and there are no significant associated harms. |
| Dissemination policy: trial results | [#31a](https://www.goodreports.org/reporting-checklists/spirit/info/#31a) | Plans for investigators and sponsor to communicate trial results to participants, healthcare professionals, the public, and other relevant groups (eg, via publication, reporting in results databases, or other data sharing arrangements), including any publication restrictions | All relevant data and report will be available online at ARI website and Clinicaltrials.gov. |  |
| Dissemination policy: authorship | [#31b](https://www.goodreports.org/reporting-checklists/spirit/info/#31b) | Authorship eligibility guidelines and any intended use of professional writers | Dr Abdul Hameed is the principal investigator and senior researcher who has drafted the protocol, study design, sampling, preparation of data tools and analysis. He has critically reviewed and revised the protocol. Daud Malik helped to critically review and revise the protocol. All authors read and approved the final protocol for publication. These two professionals have authorship. However, any Professional medical will hire for this study to improve clarity and structure in a trial report. He/She will be acknowledged in trial reports in the acknowledgement section of the report. |  |
| Dissemination policy: reproducible research | [#31c](https://www.goodreports.org/reporting-checklists/spirit/info/#31c) | Plans, if any, for granting public access to the full protocol, participant-level dataset, and statistical code | N/A | Protocol is available online at https://clinicaltrials.gov/study/NCT05715164 |
| **Appendices** | | | | |
| Informed consent materials | [#32](https://www.goodreports.org/reporting-checklists/spirit/info/#32) | Model consent form and other related documentation given to participants and authorised surrogates | INFORMED CONSENT: My name is <name of the enumerator> and I represent Alternative Research Initiative (ARI) which has been hired for the data collection for the study “Electronic Cigarettes and Nicotine Pouches for Smoking Cessation in Pakistan”. This project is related to the smoking cessation and the efficacy of smoking cessation products under the tobacco control initiatives by the ARI and Smoke-Free World, a US nonprofit, which focuses on public health. The ARI is committed to transitioning to a smoke free world, with a focus on public health, education, and knowledge creation. This interview will take 45-50 minutes, and all responses will be kept confidential. |  |
| Biological specimens | [#33](https://www.goodreports.org/reporting-checklists/spirit/info/#33) | Plans for collection, laboratory evaluation, and storage of biological specimens for genetic or molecular analysis in the current trial and for future use in ancillary studies, if applicable | N/A | Not required |

It is strongly recommended that this checklist be read in conjunction with the SPIRIT 2013 Explanation & Elaboration for important clarification on the items. Amendments to the protocol should be tracked and dated. The SPIRIT checklist is copyrighted by the SPIRIT Group under the Creative Commons “[Attribution-NonCommercial-NoDerivs 3.0 Unported](http://www.creativecommons.org/licenses/by-nc-nd/3.0/)” license. This checklist can be completed online using https://www.goodreports.org/, a tool made by the EQUATOR Network in collaboration with Penelope.ai
