# Supplementary material for: Clinical study protocol on electronic cigarettes and nicotine pouches for smoking cessation in Pakistan: a randomized controlled trial
Source: Trials. 2024 Jan 2;25:9. doi: 10.1186/s13063-023-07876-y (PMC10759381; doi:10.1186/s13063-023-07876-y)
Supplement: Supplementary file 2 — Additional file 2. [file 13063_2023_7876_MOESM2_ESM.docx]

| Data category | Information |
| --- | --- |
| Primary registry and trial identifying number | https://clinicaltrials.gov/ct2/show/NCT05715164 |
| Date of registration in primary registry | 2023-02-06 |
| Source(s) of monetary or material support | Foundation for a Smoke Free World INC |
| Primary sponsor | Foundation for a Smoke Free World INC |
| Secondary sponsor(s) | Alternative Research Initiative |
| Contact for public queries | Daud Malik (daud31us@yahoo.com) |
| Contact for scientific queries | Dr. Abdul Hameed (hameedleghari@gmail.com) |
| Public title | Electronic Cigarettes and Nicotine Pouches for Smoking Cessation |
| Scientific title | Clinical Study Protocol on Electronic Cigarettes and Nicotine Pouches for Smoking Cessation in Pakistan: A Randomized Controlled Trial |
| Countries of recruitment | Pakistan |
| Health condition(s) or problem(s) studied | Tobacco Harm Reduction Products |
| Intervention(s) | Device: E-cigarettes device plus liquid |
|  | Drug: Nicotine Pouches |
|  | Other: Basic care counseling about smoking cessation |
| Key inclusion and exclusion criteria | Participants who are at least eighteen years old.  More than 10 combustible cigarettes smoke a day at the time of study enrollment.  Smoked cigarettes for at least a year.  Participants are willing to stop combustible smoking.  Participants who can sign a written consent form.  There can only be one applicant per household.  Own a phone that supports text massaging. |
|  | Women who are pregnant.  Childbearing mothers.  Currently using other nicotine- and non-nicotine-based cessation therapies.  Expectant ladies who intend to become pregnant during the trial's participation term.  Experienced chest pain, or another cardiovascular event or procedure (e.g., heart attack, stroke, insertion of stent, bypass surgery). |
|  |  |
| Study type | Interventional |
|  | Allocation: randomized intervention model. Parallel assignment |
|  | Primary purpose: prevention |
|  |  |
| Date of first enrolment | June 2024 |
| Target sample size | 600 |
| Recruitment status | Not Recruited Yet |
| Primary outcome(s) | Number of participants reported adverse events of e-cigarettes or nicotine pouches. Change in the number of combustible cigarettes per day and point-prevalence abstinence |
| Key secondary outcomes | 7-day point-prevalence abstinence and harm reduction |
